# Can carotid artery Doppler variations induced by the end-expiratory occlusion maneuver predict fluid responsiveness in septic shock patients?

**DOI:** 10.1186/s13054-023-04422-9

**Published:** 2023-04-19

**Authors:** Sonia D’Arrigo, Antonio Maria Dell’Anna, Claudio Sandroni, Antonio Messina, Sofia Cacciola, Chiara Pacini, Massimo Antonelli

**Affiliations:** 1 Department of Anesthesia and Intensive Care, Fondazione Policlinico Universitario ‘A. Gemelli’ IRCCS, Università Cattolica del Sacro Cuore, Largo Gemelli 8, 00168 Rome, Italy; 2 Humanitas Clinical and Research Center – IRCCS, Rozzano, Milan, Italy; 3 Department of Biomedical Sciences, Humanitas University, Pieve Emanuele, Milan, Italy

**Keywords:** Fluid responsiveness, End-expiratory occlusion test (EEOt), Fluid challenge, Carotid artery Doppler, Heart–lung interaction

## Abstract

**Background:**

An increase in cardiac index (CI) during an end-expiratory occlusion test (EEOt) predicts fluid responsiveness in ventilated patients. However, if CI monitoring is unavailable or the echocardiographic window is difficult, using the carotid Doppler (CD) could be a feasible alternative to track CI changes. This study investigates whether changes in CD peak velocity (CDPV) and corrected flow time (cFT) during an EEOt were correlated with CI changes and if CDPV and cFT changes predicted fluid responsiveness in patients with septic shock.

**Methods:**

Prospective, single-center study in adults with hemodynamic instability. The CDPV and cFT on carotid artery Doppler and hemodynamic variables from the pulse contour analysis EV1000™ were recorded at baseline, during a 20-s EEOt, and after fluid challenge (500 mL). We defined responders as those who increased CI ≥ 15% after a fluid challenge.

**Results:**

We performed 44 measurements in 18 mechanically ventilated patients with septic shock and without arrhythmias. The fluid responsiveness rate was 43.2%. The changes in CDPV were significantly correlated with changes in CI during EEOt (*r* = 0.51 [0.26–0.71]). A significant, albeit lower correlation, was found for cFT (*r* = 0.35 [0.1–0.58]). An increase in CI ≥ 5.35% during EEOt predicted fluid responsiveness with 78.9% sensitivity and 91.7% specificity, with an area under the ROC curve (AUROC) of 0.85. An increase in CDPV ≥ 10.5% during an EEOt predicted fluid responsiveness with 96.2% specificity and 53.0% sensitivity with an AUROC of 0.74. Sixty-one percent of CDPV measurements (from − 13.5 to 9.5 cm/s) fell within the gray zone. The cFT changes during EEOt did not accurately predict fluid responsiveness.

**Conclusions:**

In septic shock patients without arrhythmias, an increase in CDPV greater than 10.5% during a 20-s EEOt predicted fluid responsiveness with > 95% specificity. Carotid Doppler combined with EEOt may help optimize preload when invasive hemodynamic monitoring is unavailable. However, the 61% gray zone is a major limitation (retrospectively registered on Clinicaltrials.gov NCT04470856 on July 14, 2020).

**Supplementary Information:**

The online version contains supplementary material available at 10.1186/s13054-023-04422-9.

## Background

Fluid responsiveness is defined as an increase in cardiac index (CI) in response to an increase in preload [1]. In patients with hemodynamic instability, detecting fluid responsiveness with high specificity may avoid an unnecessary fluid load, which could be associated with increased morbidity, mortality, and hospital length of stay [2, 3].

Several functional hemodynamic tests for assessing fluid responsiveness have been introduced in clinical practice in the last few years [4]. These tests measure the hemodynamic response to a transient variation in cardiac preload induced by changes in body position, as is the case of passive leg raising (PLR) [5, 6], or by changes in intrathoracic pressure induced by a transient increase in tidal volume [7, 8], a lung recruitment or sigh maneuver [9, 10], or an end-expiratory occlusion test (EEOt) [11].

The EEOt is based on an interruption of mechanical ventilation at the end of expiration for 15–30 s. This maneuver increases cardiac preload, venous return, and stroke volume (SV) in preload-responsive patients. In one study [11], an increase in CI ≥ 5% during a 15-s EEOt reliably predicted an increase in CI by > 15% in response to a 500-mL crystalloid infusion with a sensitivity and a specificity of 91% and 100%, respectively. The accuracy of EEOt in predicting fluid responsiveness is comparable to that of PLR [12]. However, detecting the rapid and transient increase in CI during the EEOt needs continuous hemodynamic monitoring. In previous studies, this has been achieved using pulse contour arterial waveform analysis [7, 11, 13–15] or changes in velocity–time integral (VTI) assessed by trans-thoracic echocardiography (TTE) [16, 17]. When invasive hemodynamic monitoring is unavailable, or TTE cannot be obtained, carotid Doppler could be a feasible alternative to track changes in SVI or CI induced by EEOt. The variations in the systolic peak velocity and the duration of the systolic component of each cardiac cycle, measured from the onset to the dicrotic notch, provide an estimate of the changes in stroke volume [18–20]. However, the potential of carotid Doppler to assess fluid responsiveness in combination with EEOt in critical care patients has not been investigated yet.

We conducted this study to assess (1) if there was a correlation between the changes in carotid Doppler peak velocity (CDPV) or in corrected flow time (cFT) and the changes in CI induced by a 20-s EEOt; (2) if EEOt-induced changes in CDPV and cFT predict fluid responsiveness in patients admitted to intensive care unit (ICU) with septic shock.

## Methods

### Patient population

The institutional review board of Policlinico Agostino Gemelli University Hospital approved the present study (Prot. N. 0048329/18 ID 1677) retrospectively registered on ClinicalTrials.gov NCT04470856 on July 14, 2020. All patients or their legal representatives gave their written informed consent to participate.

All adult patients (≥ 18 y) admitted to the general ICU of Policlinico Agostino Gemelli University Hospital with a diagnosis of septic shock who were sedated and mechanically ventilated were considered for inclusion. Patients were eligible if they needed a fluid challenge according to predefined clinical criteria: (1) hypotension (defined as a systolic arterial pressure ≤ 90 mmHg) and/or (2) tachycardia (i.e., ≥ 100 beats/min) and/or (3) urinary flow ≤ 0.5 mL/kg/min for 2 h. Those with significant valvular heart disease, cardiac arrhythmia, heart failure, peripheral arterial disease, or common carotid artery stenosis narrower than 50% were excluded from the study.

### Intervention

All patients were supine with their trunk elevated 30°, sedated, paralyzed, and mechanically ventilated in the volume control mode (Evita Infinity-V500—Drager—Italy Spa or Servo-U Maquet Medical System, Wayne, NJ, USA). We ascertained the absence of spontaneous breathing activity by evaluating the flow/pressure curves displayed on the ventilator screen. Tidal volume was set at 6 mL/kg of predicted body weight (PBW).

We measured CI using transpulmonary thermodilution (TPTD) (EV1000™/Volume View Edwards Lifesciences Corporation, Irvine, CA 92614). CI and the other hemodynamic variables derived from pulse contour analysis were recorded over 20 secs [21]. Before each measurement, we calibrated our system by injecting at least three 15-mL boluses of cold (4 °C) saline. We averaged the three measures only if none had a difference greater than 20% of the mean value [21].

EEOt consisted of a prolonged expiratory pause (20 s) in which no expiratory or inspiratory efforts were recorded.

Fluid responsiveness was defined as an increase in CI of more than 15% after a standardized fluid challenge (500 mL of Ringer’s lactate in 10 min).

Fluid responders were tested more than once, at least 12 h apart, if they met the inclusion criteria again. Each EEOt was considered a single and independent measure.

**Fig. 1:**
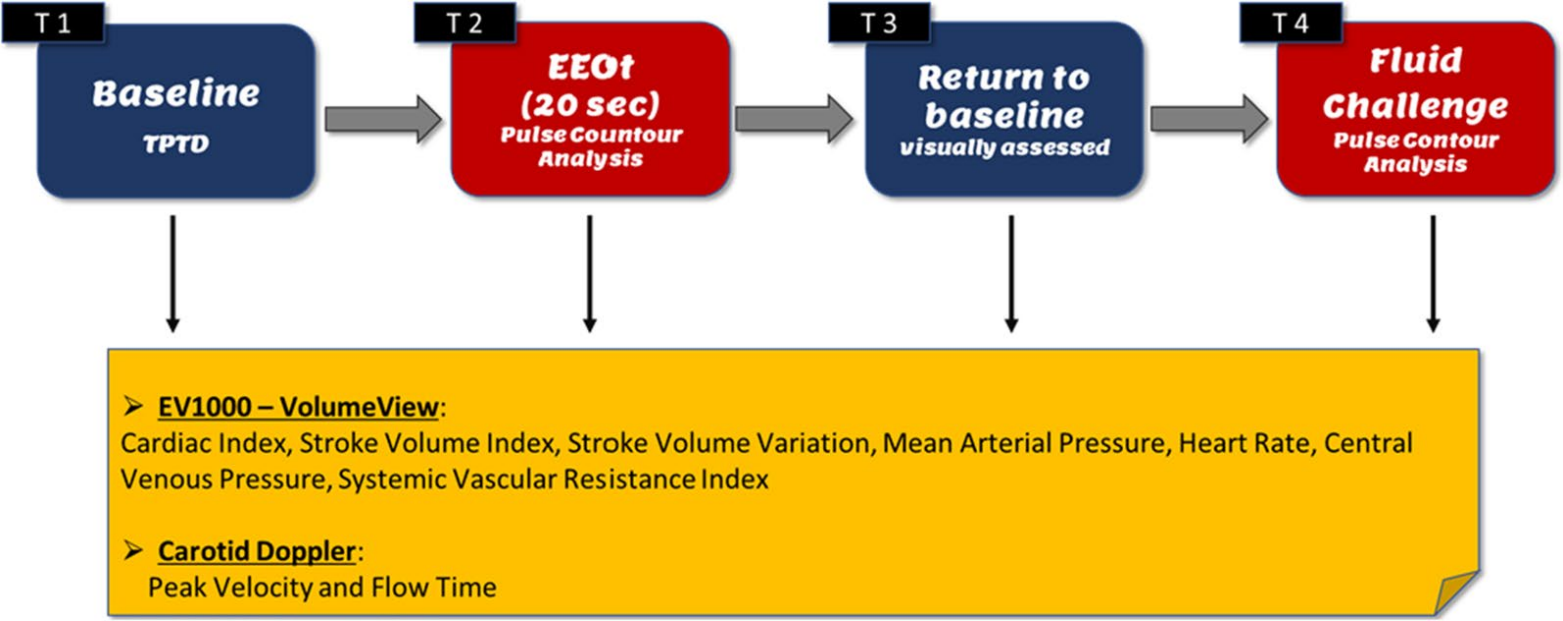
Study design

The following protocol describes our procedure in detail (Fig. 1).

*T1 (baseline 1):* we performed the first set of TPTD to assess the CI, the SVI, the stroke volume variation (SVV), and the systemic vascular resistance index (SVRI). We also recorded heart rate (HR), mean arterial pressure (MAP), and central venous pressure (CVP). Simultaneously, we measured the CDPV and the cFT using carotid Doppler (see below).

*T2 (EEOt):* one operator applied a 20-s EEOt and measured the hemodynamic variables using the pulse contour method, while a second operator performed a carotid Doppler and recorded CDPV and cFT during the last five cardiac cycles before the end of EEOt.

*T3 (baseline 2)*: we visually verified that the hemodynamic values had returned to the baseline before administering the fluid challenge.

*T4 (fluid challenge):* the patients received a 500-mL bolus of Ringer’s lactate solution in 10 min. After completing the fluid challenge, we performed the last series of hemodynamic measurements, including CI, MAP, HR, SVI, CVP, SVRI, and carotid Doppler, as above.

Two operators collected the hemodynamic variables manually and kept a screenshot of the measurements as a record for data check. Catecholamine infusion, mechanical ventilation settings, and bed position were kept constant at all times.

### Carotid Doppler

One investigator (SD'A) with previous formal training in critical care ultrasound insonated the common carotid artery in a longitudinal view approximately 1–2 cm before the bifurcation using a 5–10-MHz linear array transducer (Vivid™ iq, General Electric Healthcare, Chicago, Illinois, USA) and performed a pulse-wave Doppler analysis. The sample volume was in the vessel’s middle, with the caliper positioned parallel to flow, angled at no more than 60°. We averaged five consecutive measurements to limit the effects of respiratory-induced changes in CDPV and cFT.

The ΔCDPV was expressed as a percentage and was calculated with the following formula:

$$\left( {{\text{average}}\;{\text{CDPV}}_{{{\text{post}}}} {-}{\text{ average}}\;{\text{CDPV}}_{{{\text{pre}}}} /{\text{ average}}\;{\text{CDPV}}_{{{\text{pre}}}} } \right)*{1}00$$

where CDPV_pre_ values refer to the Baseline 1 and the CPDV_post_ values refer to those obtained during EEOt or after fluid challenge.

The FT was measured from the beginning of the flow tracing’s upstroke to the nadir of its dicrotic notch on a pulse waveform analysis. We used the Wodey’s formula: cFT = FT + 1.29*(HR-60) to correct measurements for the heart rate [22]. We measured the ΔcFT using the same formula we used for ΔCDPV.

### Study endpoints

The primary endpoint of this study was the correlation between changes in CDPV and CI during an EEOt. Secondary endpoints were the correlations between changes in CDPV and CI after the fluid challenge and between changes in cFT and CI during EEOt and after fluid challenge. We also explored the ability of changes in CDPV and cFT during EEOt to predict fluid responsiveness. Differences between responders and non-responders were studied during all three phases of our observation (baseline, EEOt, and fluid challenge).

### Statistical analysis

The distribution of continuous variables was assessed with the Kolmogorov–Smirnov test. Data are expressed as mean ± standard deviation (SD) or median (interquartile range), as appropriate. Categorical variables are reported as fractions and percentages (%). Differences between parametric variables were evaluated with the Student’s t test for unpaired and paired measures or with the Mann–Whitney and Wilcoxon tests, as appropriate. Fisher’s exact test was adopted for categorical variables. LSD and Scheffé’s test were used to correct for multiple comparisons.

We used two-way ANOVA to detect significant variable changes across time points between two groups. We calculated a receiving-operator characteristic (ROC) curve using the Wilson–Brown method to summarize the ability of each measure to predict fluid responsiveness across the various thresholds. For all predictors, we identified the optimal threshold based on the Youden index J (*J* = sensitivity + specificity − 1).

Sample size calculation for the primary outcome was made considering a correlation between CDPV changes and CI changes during EEOt of 0.8 with a null hypothesis of correlation set at 0.5. With beta error at 0.8 and a two-sided alfa error of 0.05, 38 measurements were needed to test the study hypothesis. For the area under the ROC curve (AUROC), we considered an AUROC of 0.75, a null hypothesis of 0.5, and beta and alpha error as above. The total required sample was 40 measurements.

For the carotid Doppler variables, we calculated how many measurements fell within the gray zone, i.e., the number of cases with a sensitivity < 90% and/or a specificity < 90%.

We also estimated the precision and the least significant change (LSC) [23] of CDPV. Each velocity was measured five consecutive times in each patient.

Precision was estimated as

$${2}\;{\text{SD}}/{\text{mean}}/{\text{sqrt}}\;{(}n{)}$$

where SD is the standard deviation of the measurements, and n is the number of measurements, based on the formula described by Cecconi et al. [24]

The LSC was calculated as

$${\text{precision}}*{\text{sqrt}}\;{(2)}$$

and expressed as a percentage. We performed the repeated measures correlation (rmcorr) [25] between the percentage changes in CI vs. the percentage changes in CDPV or cFT during EEOt and the percentage changes in CI vs. the percentage changes in CDPV or cFT after the fluid challenge. We stratified the results of the rmcorr for responders and non-responders. Finally, to assess the concordance between the changes of Doppler variables and the corresponding changes of CI we calculated four-quadrant clinical concordance plots [26]. In these graphs, we plotted the measurements where the changes of CDPV or cFT and the changes of CI, both expressed as a percentage, had the same direction and the same extent. These changes were divided into three categories, non-significant change (ΔCI ± 5% or less); moderate change (ΔCI ± 5–15%); or large change (ΔCI ± 15% or more). We plotted the Doppler/CI concordance during the EEOt and the fluid challenge. In addition, to illustrate the predictive ability of Doppler variables, we plotted their percentage changes at EEOt vs. the changes in CI after the fluid challenge.

We used SPSS® 28 software (IBM Chicago IL), MedCalc® Statistical Software version 20.210 (MedCalc Software Ltd, Ostend, Belgium; https://www.medcalc.org; 2022), and the rmcorr R package (https://cran.r-project.org/web/packages/rmcorr/) for statistical analysis.

## Results

We performed 44 measurements on 18 patients. Their characteristics are shown in Table 1. At inclusion, all patients were in septic shock [27] and were sedated and ventilated in volume control mode using lung-protective ventilation (mean tidal volume 6 ± 0.5 mL/kg).

**Table 1:** Patient characteristics

	Patients (*n* = 18)
Age, years	64 ± 10
Males	14 (78)
Body mass index (Kg/m^2^)	27 ± 5
SAPS II score	60 [46–81]
SOFA score at inclusion	9.5 [8–12]

	All measurements (*n* = 44)
Norepinephrine dose (mcg/Kg/min)	0.7 ± 0.6
PEEP (cmH_2_O)	6 ± 2
Compliance (mL/cmH_2_O)	38 ± 9
Driving pressure (cmH_2_O)	13 ± 3
Plateau pressure (cmH_2_O)	19 ± 4
Tidal volume (mL/Kg)	6 ± 0.5
Respiratory rate (breaths/min)	20 ± 3

Data are expressed as median [IQR], mean ± standard deviation, or n (%)

PEEP, positive end-expiratory pressure; SAPS, Simplified Acute Physiology Score; and SOFA, Sequential Organ Failure Assessment

The overall rate of fluid responsiveness was 43.2% (19 out of 44 measures).

### Baseline

At baseline, fluid responders had a significantly lower CI (2.2 ± 0.6 vs. 2.8 ± 0.9 L/min/m^2^; *p* = 0.012) and a higher SVV (13.7 ± 6.4 vs. 10.0 ± 3.4%; *p* = 0.029) than non-responders. There were no significant differences in HR, MAP, CVP, and SVRI. Fluid responders also had a significantly lower CDPV (88.1 ± 24.6 vs. 111.2 ± 37.5 cm/s, *p* = 0.019) and cFT (266.3 ± 31.0 vs. 285.1 ± 24.7 ms, *p* = 0.037) than non-responders (Table 2).

**Table 2:** Hemodynamic variables at baseline, during EEOt and after fluid challenge in responders (*n* = 19) and non-responders (*n* = 25)

Variables	Baseline	EEOt	After fluid challenge
*HR (bpm)*			
Responders	89.8 ± 16.0	88.6 ± 15.1	84.3 ± 15.0
Non-responders	96.4 ± 21.2	95.4 ± 21.2	90.5 ± 21.4
*MAP (mmHg)*			
Responders	66.5 ± 18.5	67.2 ± 16.0	78.5 ± 22.9
Non-responders	71.7 ± 12.5	72.0 ± 13.1	76.7 ± 14.3
*CI (L/min/m*^*2*^*)*			
Responders	2.2 ± 0.6*	2.5 ± 0.8	2.8 ± 0.9^§^
Non-responders	2.8 ± 0.9	2.8 ± 0.9	2.9 ± 0.9
*SVV (%)*			
Responders	13.7 ± 6.4*		9.6 ± 5.3^§^
Non-responders	10.0 ± 3.4		8.4 ± 3
*SVI (mL/b/m*^*2*^*)*			
Responders	25.1 ± 7.4*	28.3 ± 8.8	34.1 ± 9.8^§^
Non-responders	29.3 ± 7.7	29.9 ± 7.9	32.4 ± 8.1
*CVP (mmHg)*			
Responders	7.2 ± 3.7	6.6 ± 3.6	9.7 ± 4.4^§^
Non-responders	7.8 ± 5.3	7.4 ± 5.2	10.0 ± 5.8
*SVRI (dyn·s·cm*^*−5*^*)*			
Responders	2327 ± 1027	2168 ± 966	2028 ± 931
Non-responders	2038 ± 923	2016 ± 856	1951 ± 935
*CDPV (cm/s)*			
Responders	88.1 ± 24.6*	100.0 ± 34.5	108.0 ± 35.4
Non-responders	111.2 ± 37.5	113.6 ± 39.9	115.3 ± 43.7
*cFT (msec)*			
Responders	266.3 ± 31.0*	282.0 ± 30.6	292.0 ± 33.6
Non-responders	285.1 ± 24.7	292.3 ± 22.2	295 ± 30.8

Values are expressed as mean ± standard deviation

Patients were considered responders if their cardiac index increased by ≥ 15% after the 500-mL fluid challenge

*p < 0.05 for differences between responders and non-responders at baseline

^§^*p* < 0.05 for comparison between EEOt and after fluid challenge in responders

### Hemodynamic effects of EEOt

**Fig. 2:**
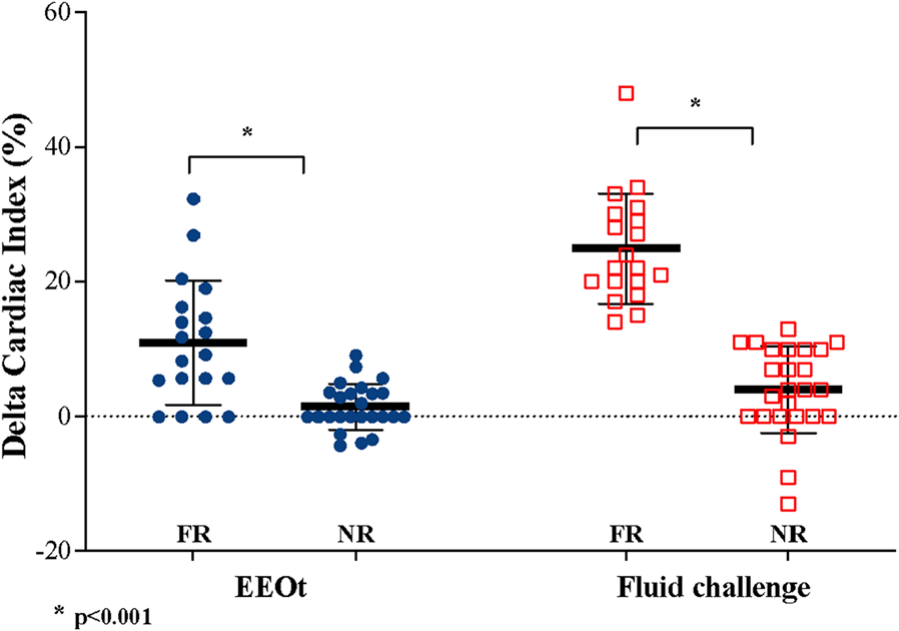
Changes in cardiac index in responders (FR) and non-responders (NFR) during EEOt and after fluid challenge

**Fig. 3:**
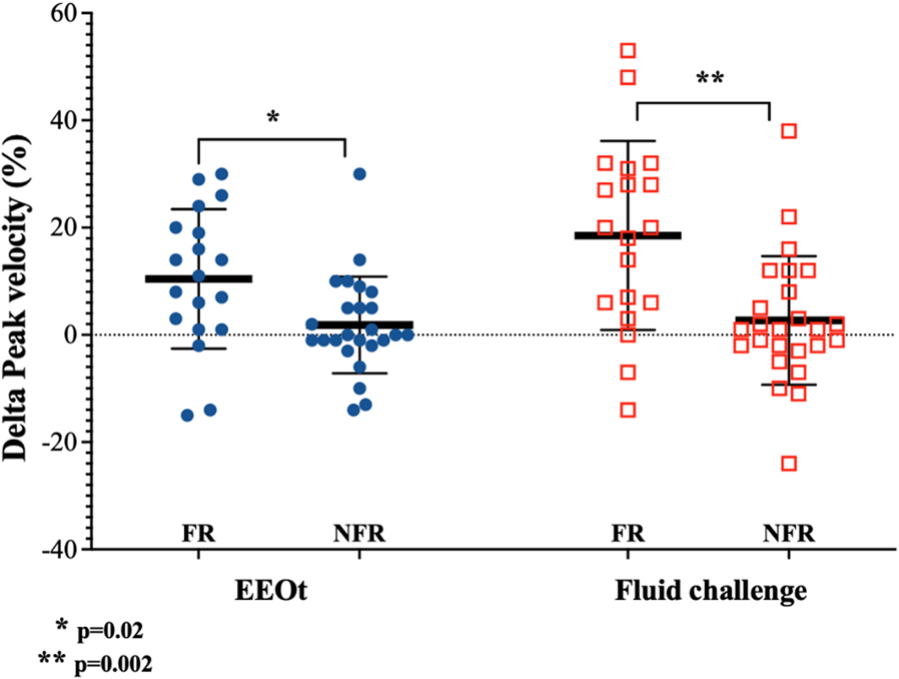
Changes in carotid Doppler peak velocity (CDPV) in responders (FR) and non-responders (NFR) during EEOt and after fluid challenge

During the EEOt, the CI increased by 10.9% (from 2.2 ± 0.6 to 2.5 ± 0.8 L/min/m^2^; *p* < 0.001) and the CDPV increased by 10.5% (from 88.1 ± 24.6 to 100 ± 34.5 cm/s; *p* < 0.05) in responders, while no significant change was observed in non-responders (Table 2 and Figs. 2, 3). The cFT showed a smaller increase that did not significantly differ between responders and non-responders (Table 2 and Additional file 1: Figure E1).

### Hemodynamic effects of fluid challenge

After the 500-mL fluid challenge, in fluid responders, the CI increased by 22.2% (from 2.2 ± 0.6 to 2.8 ± 0.9 L/min/m^2^; *p* < 0.05) and the CDPV increased by 19.6% (from 88.1 ± 24.6 to 108 ± 35.4 cm/s; *p* = 0.002), while no significant change was observed in non-responders (Table 2 and Figs. 2, 3). The cFT showed a larger increase from the baseline after the fluid challenge than during the EEOt. However, this increase did not significantly differ between responders and non-responders (Table 2 and Additional file 1: Figure E1).

The mean values of MAP, HR, SVV, CVP, and SVRI did not change significantly during EEOt and after the fluid challenge.

### Correlation between changes in carotid Doppler and in cardiac output measurements

The linear correlation coefficient r between the percentage changes in CDPV and CI during EEOt was 0.52 [0.26–0.71]. A significant but lower correlation was found for cFT (*r* = 0.35 [0.06–0.58]) (Additional file 1: Figure E2).

On rmcorr, the percentage changes in CDPV vs. the percentage changes in CI were significantly correlated across EEOt and fluid challenge in responders (*r* = 0.405 [0.046–0.733]), while no significant correlation was found in non-responders (*r* = 0.268 [− 0.147–0.600]; Additional file 1: Figure E3). Similar results were observed with cFT (*r* = 0.628 [0.257–0.838] and 0.148 [− 0.262–0.520], respectively; Additional file 1: Figure E4).

The clinical concordance plot showed that the overall fraction of concordant changes (CDPV or cFT vs. CI) was greater for cFT than for CDPV at EEOt (Additional file 1: Figure E5 A). However, the number of concordant changes above 5% was proportionally higher for CDPV. This difference was more evident in measurements performed after the fluid challenge and when comparing changes in Doppler variables at EEOt vs. changes in CI at the fluid challenge (Additional file 1: Figure E5 B–C).

### Prediction of fluid responsiveness

**Fig. 4:**
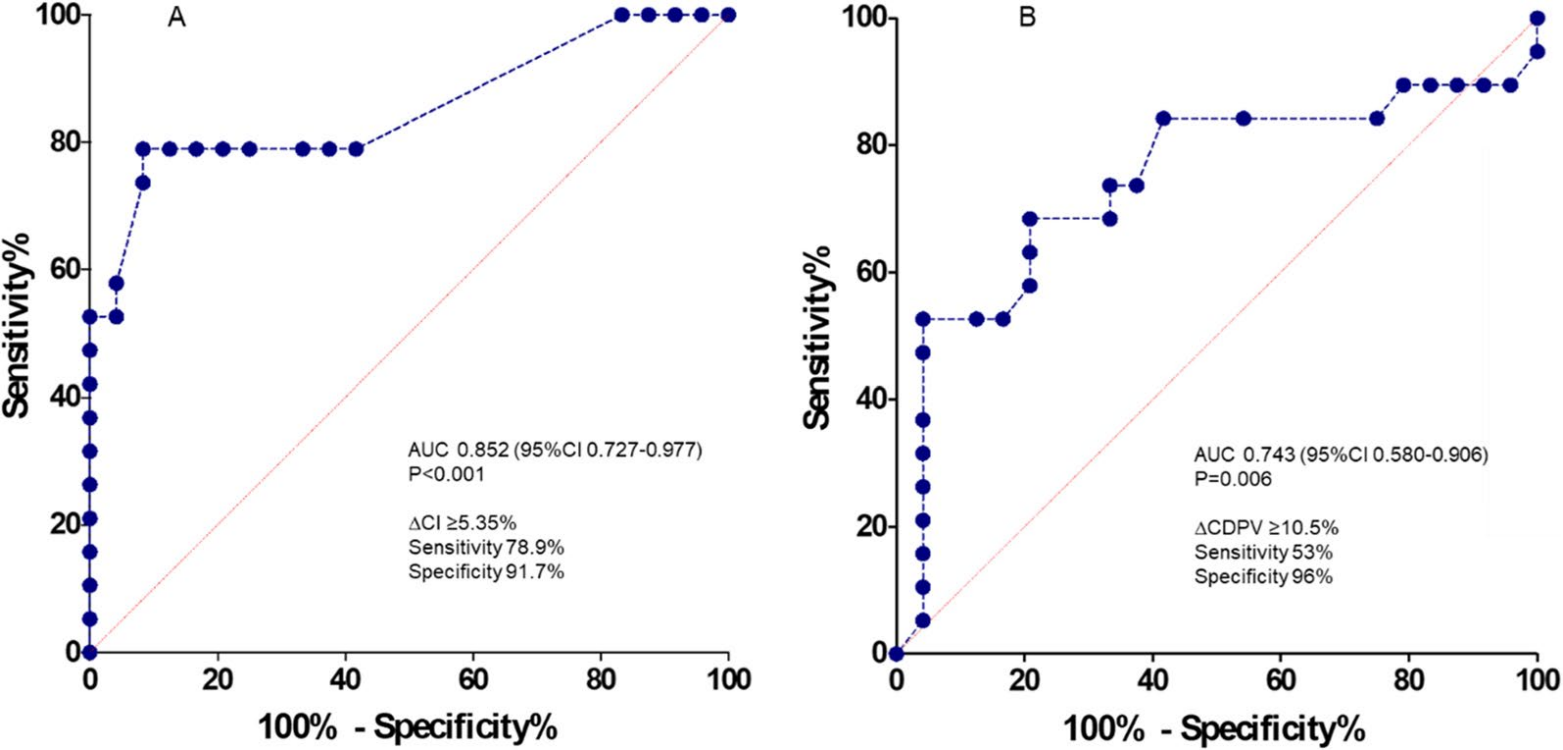
**A**, **B** ROC curves of EEOt-induced changes in CI (**A**) and CDPV (**B**) for predicting fluid responsiveness

During EEOt, an increase in CI ≥ 5.35% predicted fluid responsiveness with 91.7% specificity and 78.9% sensitivity. The AUROC of CI was 0.852 (95% CI 0.727–0.977, *p* = 0.0001) (Fig. 4A).

The LSC of carotid Doppler measures in our population was 7.4% for CDPV and 3.5% for cFT. An increase in CDPV ≥ 10.5% during EEOt predicted fluid responsiveness with 96.2% specificity (95% CI 80.0–99.8) and 53.0% sensitivity (95% CI 32.1–73.3), with an AUROC of 0.743 (95% CI 0.580–0.906, *p* = 0.006) (Fig. 4B). Sixty-one percent of measurements fell within the gray zone (CDPV ranging from − 13.5 and 9.5 cm/s). The cFT changes during EEOt did not accurately predict fluid responsiveness (AUROC 0.655 [0.496 to 0.791], *p* = 0.07; Additional file 1: Figure E6).

## Discussion

Our results showed a significant correlation between changes in CPDV during EEOt and changes in CI during the subsequent fluid challenge. An increase in CDPV greater than 10.5% during an EEOt predicted fluid responsiveness in ICU patients with septic shock with high specificity. This may allow clinicians to non-invasively identify about 40% of fluid responders and safely administer them fluids without risking a fluid overload.

The carotid artery Doppler assessment of fluid responsiveness coupled with an EEOt can be performed in mechanically ventilated patients in whom continuous cardiac monitoring is unavailable or the transthoracic echocardiographic windows are technically impaired. These conditions may be common not only in the ICU, but also in the operating room in patients undergoing major surgery whose chest wall is inaccessible to echocardiography. In this setting, the carotid artery might be the only accessible large-size arterial vessel for performing arterial Doppler. The position of the carotid artery, which is located just distally to the aortic outflow tract, makes its flow a good approximation of the aortic flow [28]. Carotid Doppler does not suffer from the technical limitations of echocardiography, where measurement of left ventricular outflow tract velocities for the estimation of SV requires specific training for adequate performance, and it is not easily obtainable, especially in ventilated patients.

To the best of our knowledge, this is the first study assessing fluid responsiveness using the carotid Doppler variations in association with an EEOt in critically ill patients. The potential usefulness of the changes in carotid Doppler during EEOt has been previously investigated in healthy volunteers [28]. The authors found a good correlation between changes in carotid VTI and aortic VTI (*ρ* = 0.79) or SV (*ρ* = 0.95). However, the results of that study are not directly applicable to the critical care setting. Moreover, that study used a different carotid Doppler equipment (wearable device) and evaluated a different index (changes in carotid VTI pattern) compared to our study.

Two studies [18, 19] investigated the accuracy of changes in carotid Doppler induced by intrathoracic pressure variation during lung-protective mechanical ventilation with low tidal volume (6 mL/kg of PBW). One study [18] measured CDPV in patients undergoing elective coronary artery bypass surgery, while the other study measured CDPV in patients with septic shock in the ICU [19]. In these studies, the AUROC of CDPV was 0.85 [0.72–0.97] and 0.88 [0.77–0.95], respectively, which is greater than the 0.74 value we found in our study. These measurements, however, were made during the respiratory cycle. Conversely, we investigated the changes between CDPV before and during the EEO maneuver.

EEOt and fluid challenge were not consistently associated with increased Doppler velocity. Namely, CDPV decreased in a minority of responders (3/19 measurements during the EEOt and 2/19 measurements after the fluid challenge) and almost half of the non-responders (11/25 measurements at either time point; Additional file 1: Figure E7). During the EEOt, the CDPV percentage decrease was below the LSC in all but five measurements (two in responders and three in non-responders). In non-responders, the CDPV changes were symmetrically distributed in both directions, which suggests a random variation. The reason for the occasional CDPV decrease we observed in fluid responders is unclear, but a patient-specific bias cannot be excluded since all the relevant measurements were obtained from a single patient.

Results of the repeated measures correlation clearly showed that the changes in CDPV and cFT both during EEOt and after fluid challenge were correlated with the parallel changes in CI or SVI in responders. This is consistent with the ability of EEOt-induced changes in CPDV to predict fluid responsiveness in our study. However, the accuracy of CDPV was lower than that of other indices, such as PLR. In fact, the gray zone of CPDV was 61%, meaning that only the CDPV values included in the remaining 39% range were sufficiently accurate to make a clinical decision. A possible explanation for this finding may be that the EEOt causes smaller changes in venous return compared to the PLR. The PLR in fact causes a rapid displacement of a high volume (about 300 mL) of venous blood from the lower body toward the right heart, mimicking an entire fluid challenge [29]. Another explanation is that we measured CDPV and CI during slightly different time windows. While the five carotid Doppler curves were recorded during the last seconds of the EEOt, when the maneuver’s effect was maximal, the SVI and CI were calculated over a 20-s time window that only partially coincided with the last seconds of the end-occlusion. A further explanation could be that the lung-protective ventilation strategy we adopted (tidal volume 6 mL/kg) may have reduced the hemodynamic impact of EEOt. However, while some studies showed a greater diagnostic accuracy of the EEOt at 8 mL/kg vs. 6 mL/kg of PBW [7, 8], other studies showed a high accuracy of EEOt at a tidal volume of 6–7 mL/kg of PBW [16, 30, 31]. In addition, a recent systematic review and meta-analysis [32] showed no significant difference between the accuracies of EEOt in studies where the tidal volume was ≤ 7 mL/kg and those using higher tidal volumes (summary AUROC 0.96 [0.92–0.97] vs. 0.89 [0.82–0.95], respectively; *p* = 0.44). Regarding PEEP, one study showed that PEEP levels ranging from 5 to 14 cmH_2_O do not affect the predictive ability of the EEOt [14]. The overall evidence suggests that ventilator settings are unlikely to affect the accuracy of EEOt.

The clinical concordance analysis of the percentage changes in Doppler variables vs. the percentage changes in CI showed that many of the concordant changes in cFT were ± 5% or less. Compared to cFT, CDPV had a greater proportion of clinically significant concordant changes (5–15% or > 15%; categories 2 and 3 in Additional file 1: Figure E5) [26]. This was evident especially when comparing the changes in Doppler variables at EEOt with the CI changes after the fluid challenge and is consistent with the greater ability of CDPV changes to predict fluid responsiveness.

Despite a clear correlation between cFT and CI changes at EEOt and fluid challenge, cFT did not show a significant predictive ability for fluid responsiveness in our study. The reasons for this are unclear and deserve further investigation. Since the LSC of cFT was only about half the percentage increase in cFT during EEOt or after the fluid challenge in fluid responders, this result does not seem to be due to an insufficient precision of the Doppler technique. Because of the small AUROC of cFT, our study was not powered enough to assess its predictive ability with confidence.

In our study, we defined fluid responsiveness as a ≥ 15% increase in CI. This choice was based on previous literature on EEOt. However, an SVI increase could have been an equally valuable target [18–20]. Carotid Doppler surrogates are more physiologically related to stroke volume than cardiac output. Moreover, stroke volume is the closest clinically available approximation of the cardiac length–tension relationship, which makes it very suitable for evaluating fluid responsiveness [33].

We may have considered adding an end-inspiratory to the end-inspiratory occlusion maneuver for predicting fluid responsiveness. In a study by Jozwiak et al. [16], the diagnostic threshold of this maneuver was 13%, higher than the 10% LSC of VTI used in that study. However, the LSC of CPDV and cFT we used in our study was lower, 7.4% and 3.5%, respectively. Both were lower than the 10.5% diagnostic threshold we found, which makes the need for adding an end-inspiratory occlusion test less stringent.

Fluid responsiveness depends on the slope of the Frank–Starling curve, which is critically affected by cardiac contractility. In our study, we excluded patients with cardiac failure, so that the role of cardiac contractility was not specifically assessed. However, previous observations made using aortic Doppler [34] have shown that CDPV is directly correlated with cardiac inotropy, while cFT is inversely correlated with systemic vascular resistance (*r*^2^ = 0.66). Carotid Doppler could therefore be used to investigate the relationship between cardiac contractility and vascular resistance in response to therapy, assuming that CDPV measurement is accurate and that blood flow in the supra-aortic vessels mirrors the blood flow in the thoracic aorta.

Some limitations of our study must be acknowledged. Firstly, this study was conducted on adult patients with septic shock in controlled ventilation with 6 mL/kg of PBW with absent or minimal spontaneous breathing, and its findings are not necessarily generalizable to other patient populations. Secondly, this technique is based on manual detection and—as such—is prone to intra-observer variation. In our study, all carotid Doppler measurements were made by a single experienced operator. While this was essential to ensure the reproducibility of our measurements, it may have overestimated their precision and reduced the external validity of our findings. Thirdly, we used the EV1000™/Volume View device, which calculates SVI and CI on the average pulse contour recorded over 20 s. Inevitably, this time window only partially overlapped with those of the EEOt, which may have affected the accuracy of our findings. Fourthly, as specified in our Methods, we compared both the hemodynamic measurements made during EEOt and those made after the fluid challenge with the first baseline (T1), since on T3 the return to the baseline after the EEOt was assessed only visually. Although the effect of the EEOt is assumed to be transient, we cannot exclude that some residual effects may have persisted after the end of EEOt and have reduced the accuracy of the subsequent measurements. Fifthly, the 61% gray zone we found in our study suggests caution when using Doppler results to make clinical decisions. Lastly, carotid Doppler might be less accurate in patients affected by arrhythmias, such as atrial fibrillation, which are common in septic shock patients [35], or by valvular disorders, severe heart failure, carotid stenosis > 50% or in some surgical settings such as brain or neck surgery.

## Conclusions

Our study showed that in adult patients with septic shock, the changes in CPDV and cFT during EEOt on carotid Doppler are correlated with the changes in CI that occur after a fluid challenge, and that an increase in CDPV greater than 10.5% during a 20-s EEOt predicted fluid responsiveness with > 95% specificity. Carotid Doppler can be used to assess fluid responsiveness in patients who are sedated and mechanically ventilated and without arrhythmias, when invasive hemodynamic monitoring is not immediately available. However, its large gray zone is an important limitation.

## Supplementary Information

**Additional file 1**. Supplementary figures.

## Data Availability

All data generated or analyzed during this study are included in this article. The dataset used and/or analyzed during the study is available from the corresponding author on request.

## Abbreviations

cFT: Corrected flow time
CI: Cardiac index
CDPV: Carotid Doppler peak velocity
CIs: Confidence intervals
CVP: Central venous pressure
ELWI: Extravascular lung water index
EEOt: End-expiratory occlusion test
FHT: Functional hemodynamic tests
GEDVI: Global end-diastolic volume index
HR: Heart rate
ICU: Intensive care unit
MAP: Mean arterial pressure
PLR: Passive leg raising
rmcorr: Repeated measures correlation
SV: Stroke volume
SVI: Stroke volume index
SVRI: Systemic vascular resistance index
SVV: Stroke volume variation
TPTD: Trans-pulmonary thermodilution
TTE: Trans-thoracic echocardiography
VTI: Velocity–time integral
