# Supplementary material for: Can carotid artery Doppler variations induced by the end-expiratory occlusion maneuver predict fluid responsiveness in septic shock patients?
Source: Crit Care. 2023 Apr 19;27:153. doi: 10.1186/s13054-023-04422-9 (PMC10116770; doi:10.1186/s13054-023-04422-9)
Supplement: Supplementary file 1 — Additional file 1. Supplementary figures. [file 13054_2023_4422_MOESM1_ESM.docx]

**Electronic Supplementary Material**


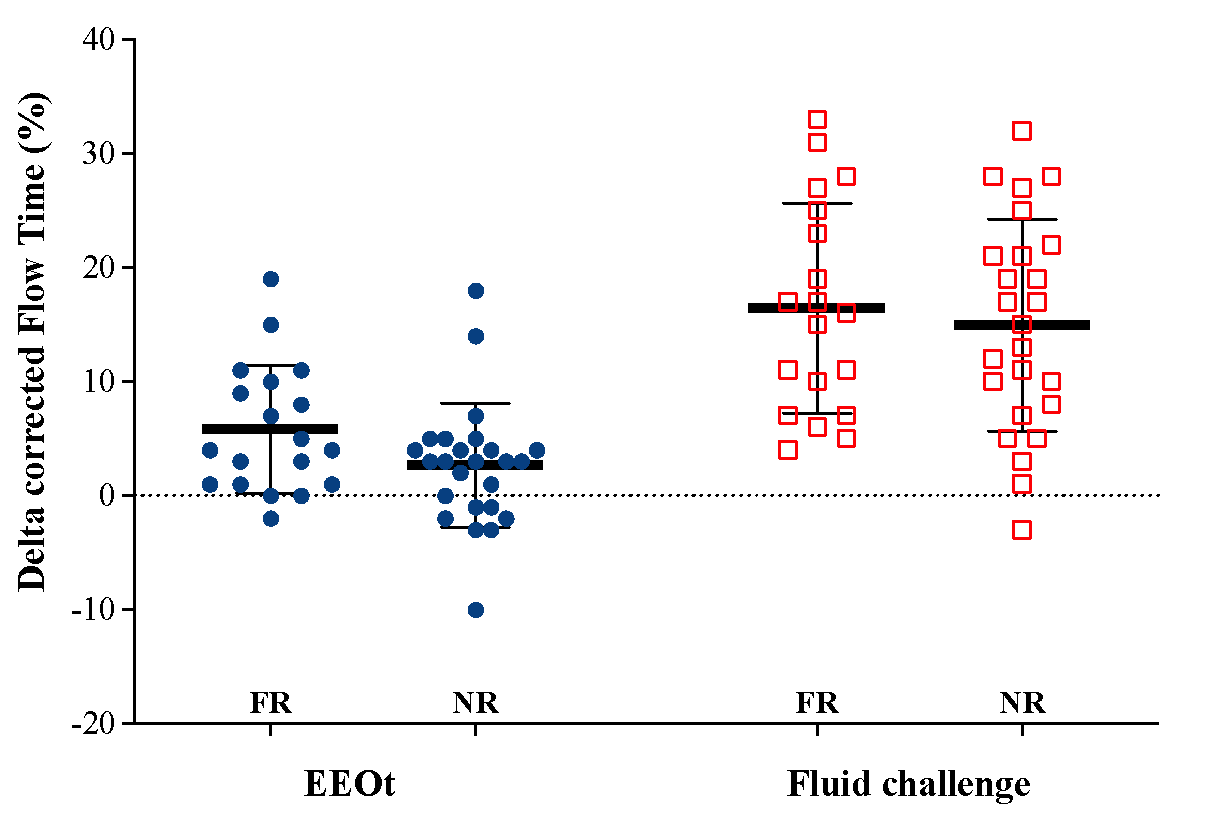


**ESM Figure E1**. Percentage difference in corrected flow time in responders (FR) and non-responders (NFR) during EEOt and after fluid challenge**.**

**A**


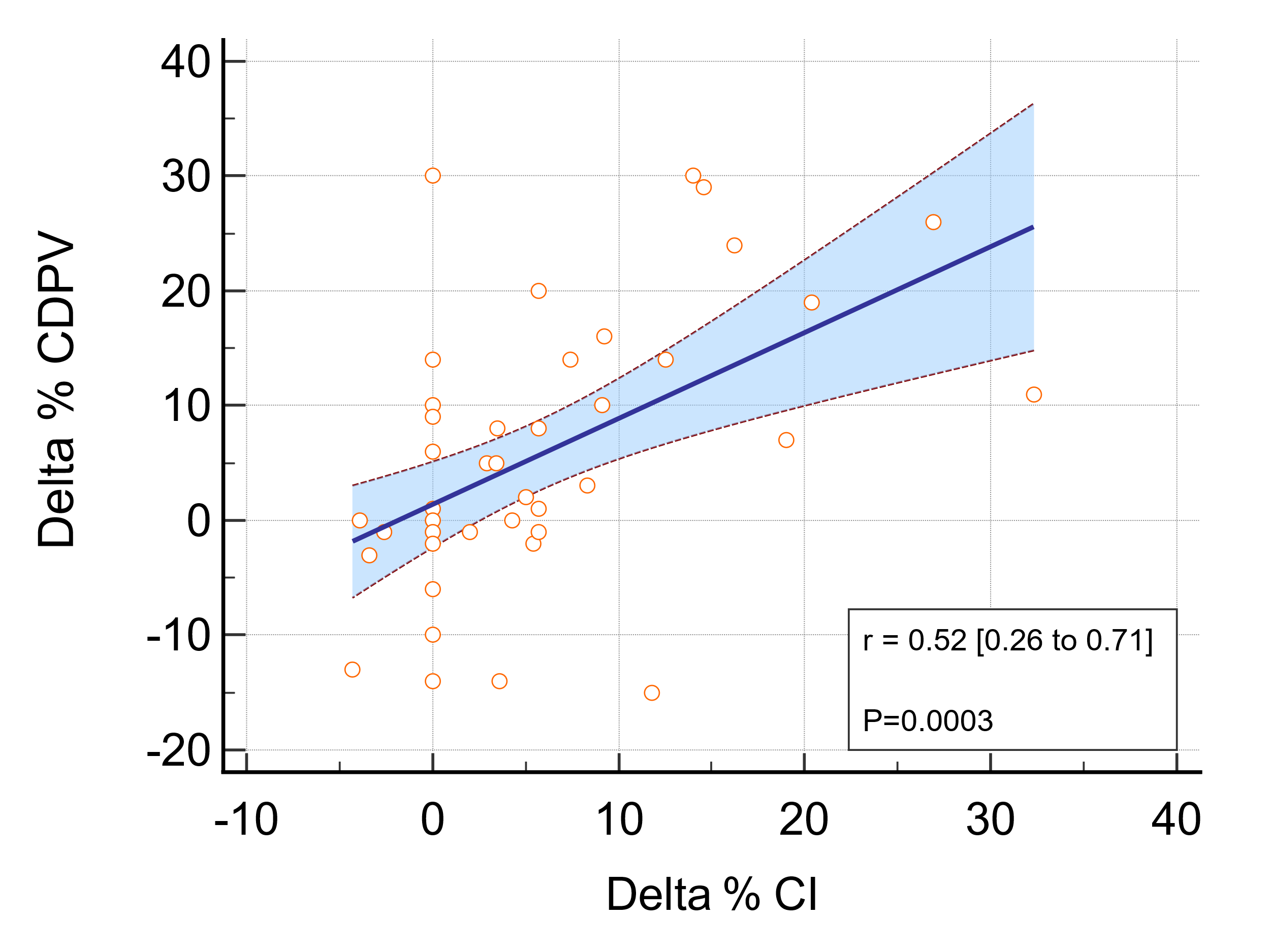


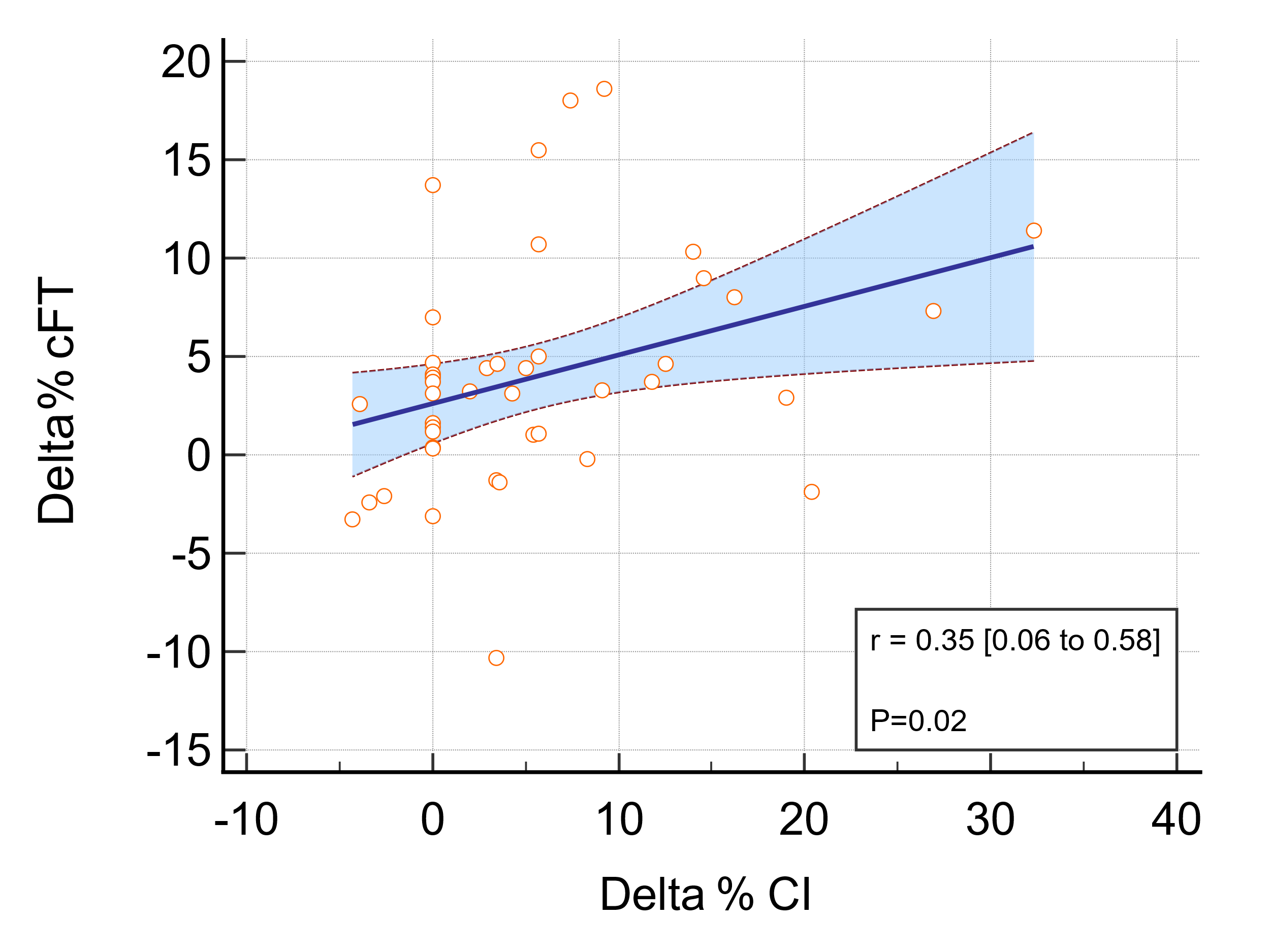


**B**

**ESM Figure E2.** Linear correlation between the percentage changes in CDPV (**A**) or cFT (**B**) and percentage changes in CI during the EEOt.

| **CDPV** | |
| --- | --- |
| 1. **Responders** | 1. **Non-responders** |
| 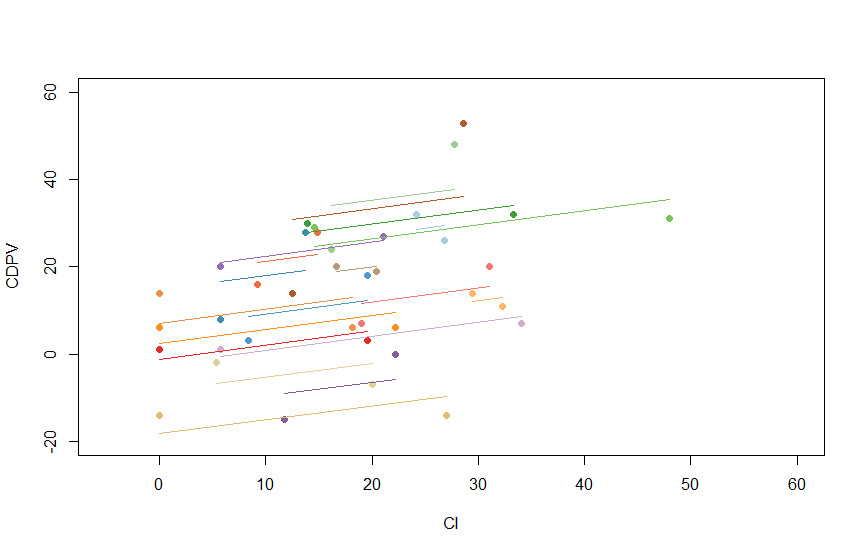 | 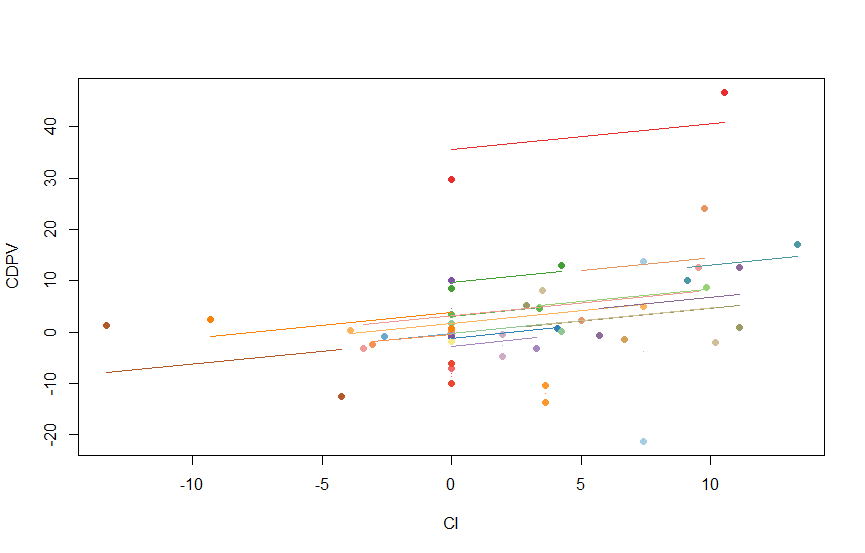 |
| r = 0.405* [95%CI = 0.046; 0.733] | r = 0.268 [95%CI =-0.147; 0. 600] |

**ESM Figure E3.** Repeated measures correlation for CDPV in responders (left chart: **A**) and non-responders (right chart: **B**). In each chart, the first data point corresponds to the correlation between the percentage change in CDPV and the percentage change in CI at EEOt. The second data point corresponds to the correlation between the percentage change in CDPV and the percentage change in CI at the fluid challenge. Each patient (or measurement) corresponds to a different color.

| **cFT** | |
| --- | --- |
| 1. **Responders** | 1. **Non-responders** |
| 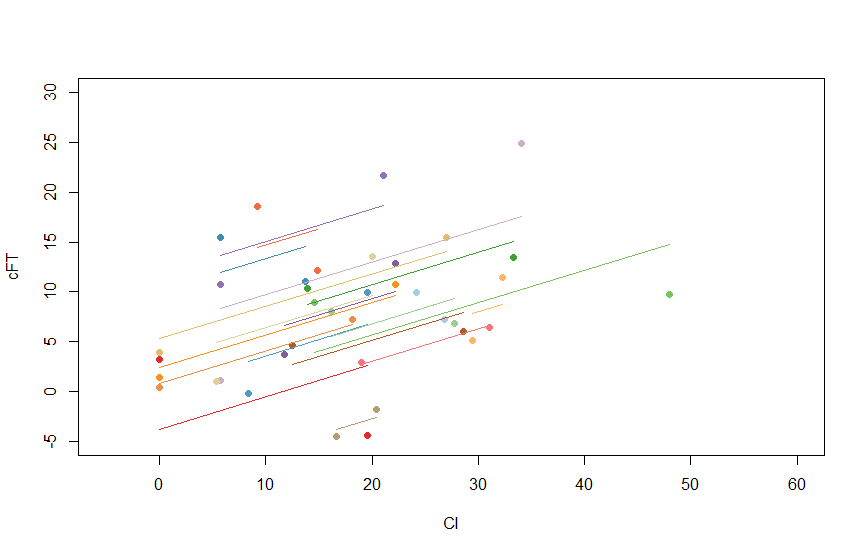 | 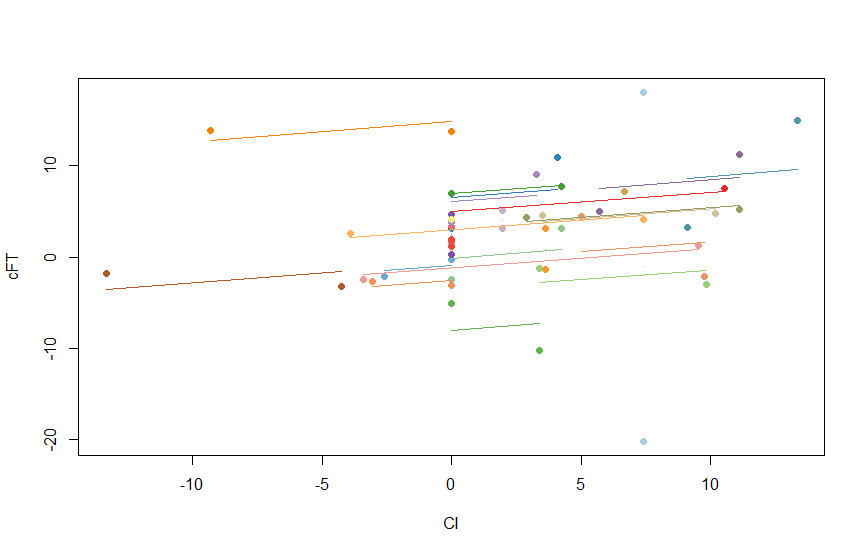 |
| r= 0.628** [95%CI = 0.257; 0.838] | r = 0.148 [95%CI = -0.262; 0.520] |

**ESM Figure E4**. Repeated measures correlation for cFT in responders (left chart: **A**) and non-responders (right chart: **B**). In each chart, the first data point corresponds to the correlation between the percentage change in cFT and the percentage change in CI at EEOt. The second data point corresponds to the correlation between the percentage change in cFT and the percentage change in CI at the fluid challenge. Each patient (or measurement) corresponds to a different color.

| **A. During EEOt** | |
| --- | --- |
| 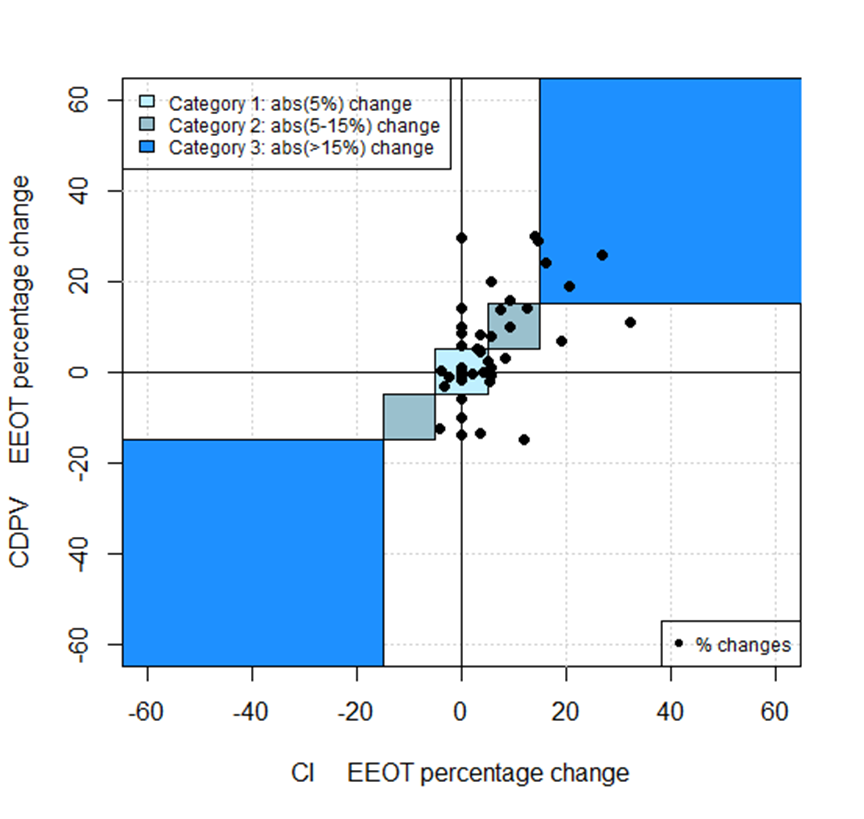 | 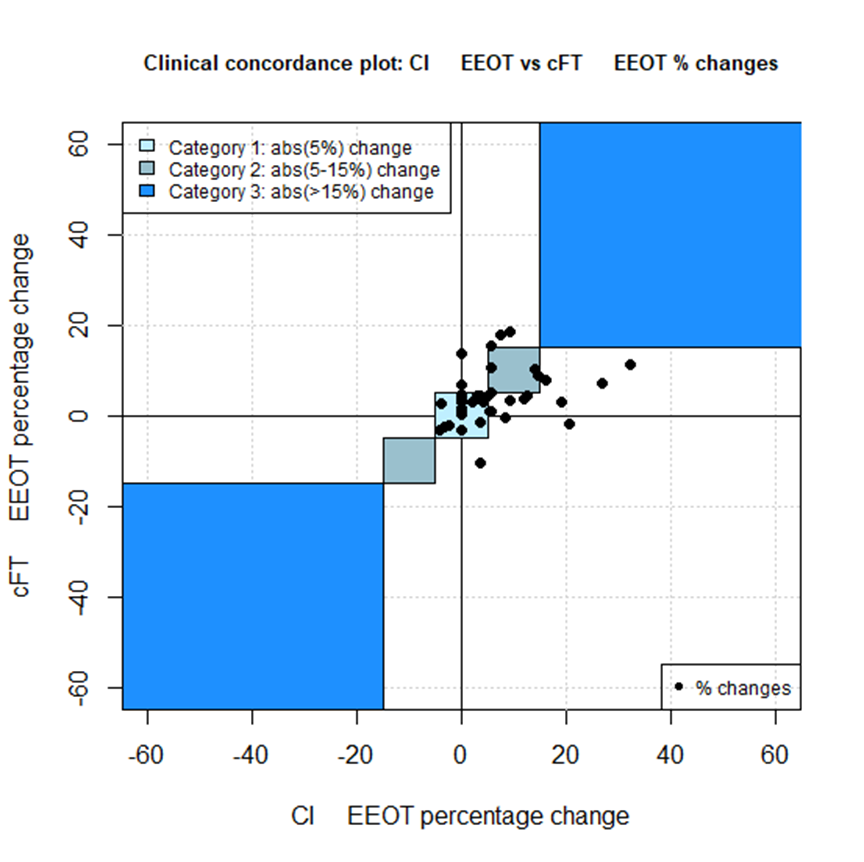 |
| Category 1: 32%  Category 2: 9%  Category 3: 7% | Category 1: 52%  Category 2: 9%  Category 3: 0% |
| **B. After fluid Challenge** | |
| 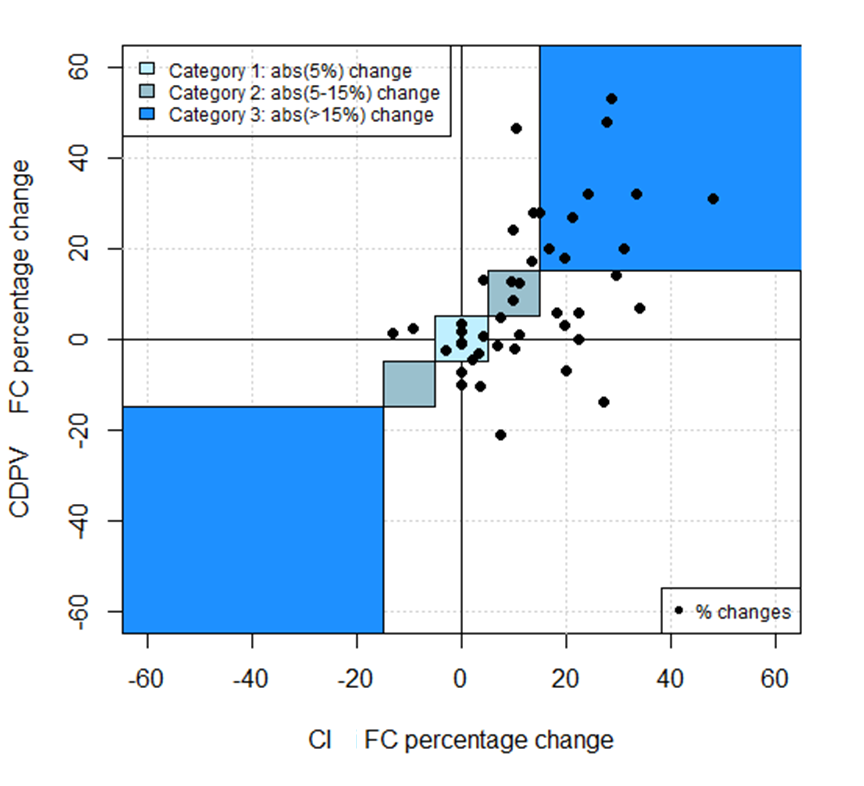 | 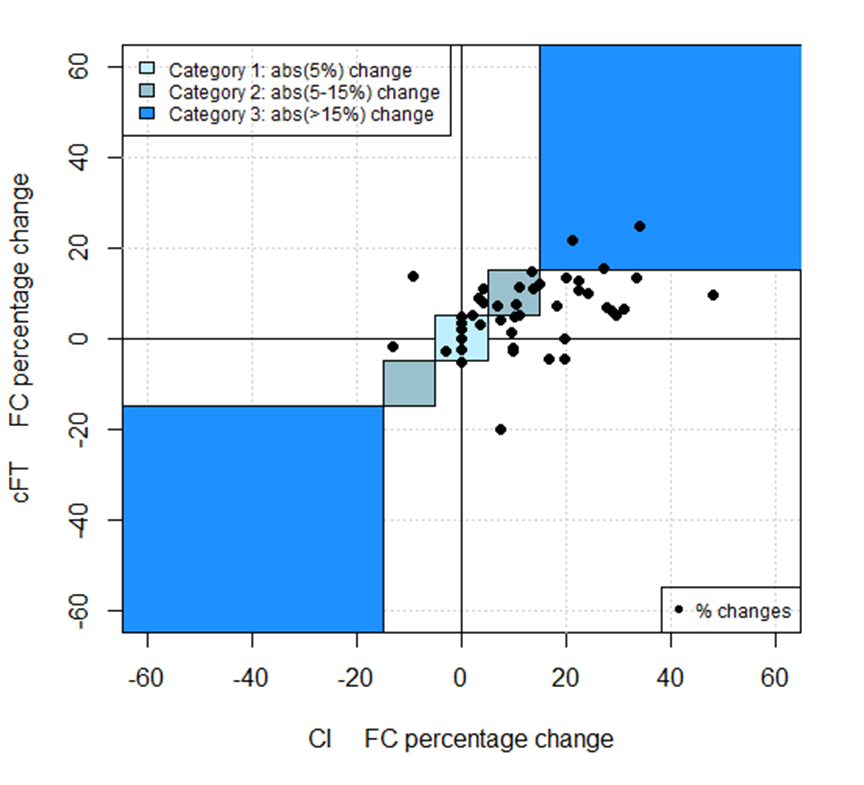 |
| Category 1: 18%  Category 2: 7%  Category 3: 20% | Category 1: 16%  Category 2: 16%  Category 3: 7% |

| **C. Changes in cFT or CDPV during EEOt vs. changes in CI after fluid challenge** | |
| --- | --- |
| 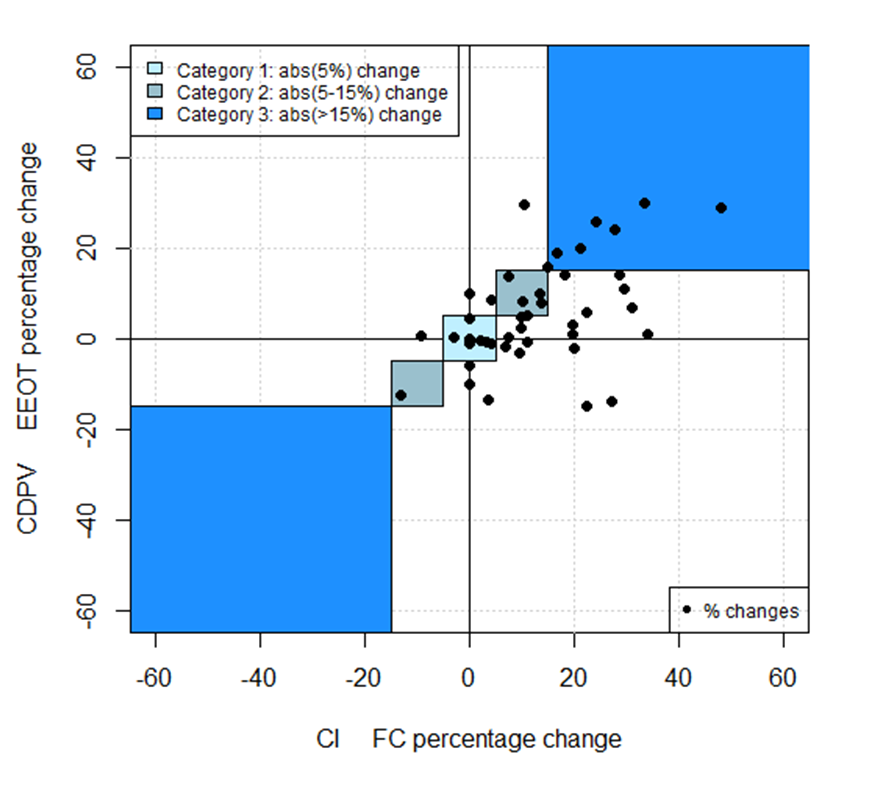 | 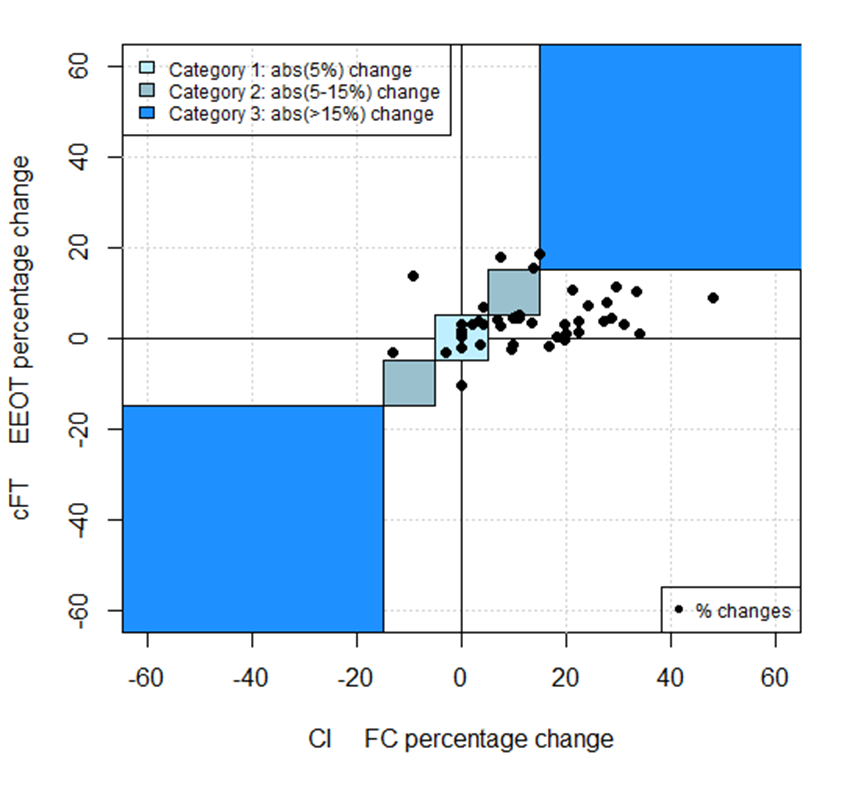 |
| Category 1: 16%  Category 2: 14%  Category 3: 14% | Category 1: 23%  Category 2: 2%  Category 3: 0% |

**ESM Figure E5**. Four-quadrant plots showing clinical concordance between the percentage change in CDPV (left) or cFT (right) vs. the percentage change in CI during EEOt (**A**) and after fluid challenge (**B**). The plots in chart **C** show the concordance between the percentage change in cFT or CDPV during EEOt vs. the percentage change in CI after a fluid challenge.

The data points represent measurements where the percentage change of Doppler variables had the same direction and extent. Changes are divided into the following categories: *Category 1* (non-significant change)*:* ∆CI ± 5% or less. *Category 2* (moderate change): ∆CI 5-15%. *Category 3*: ∆CI 15% or more. Per each category, the percentage of points over the total is reported. The remaining percentages in each plot correspond to measurements where the changes in Doppler variables or CI had not the same direction and/or extent (data points not shown).


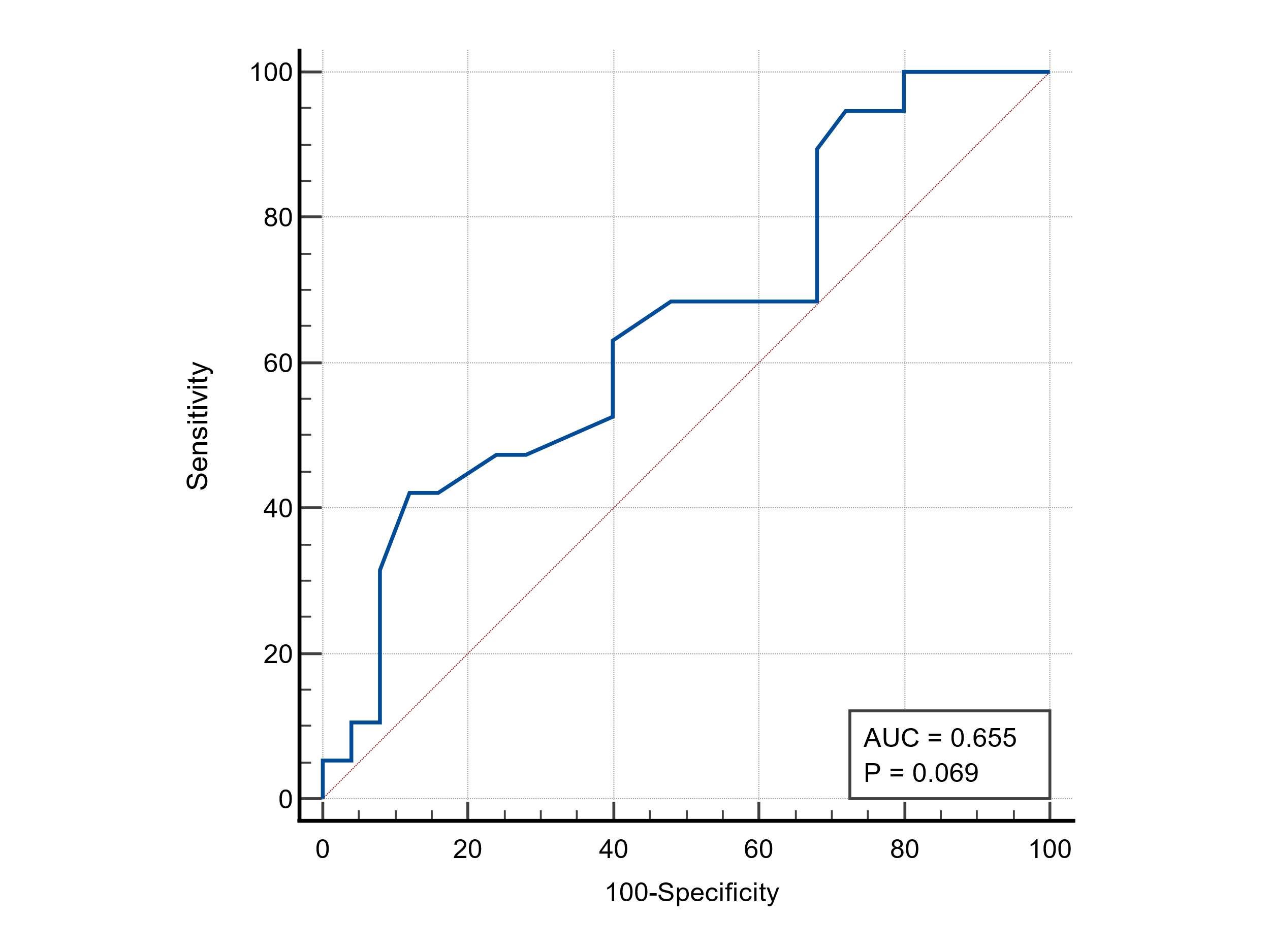


**ESM Figure E6.** ROC curve of EEOt-induced changes in cFT for predicting fluid responsiveness.


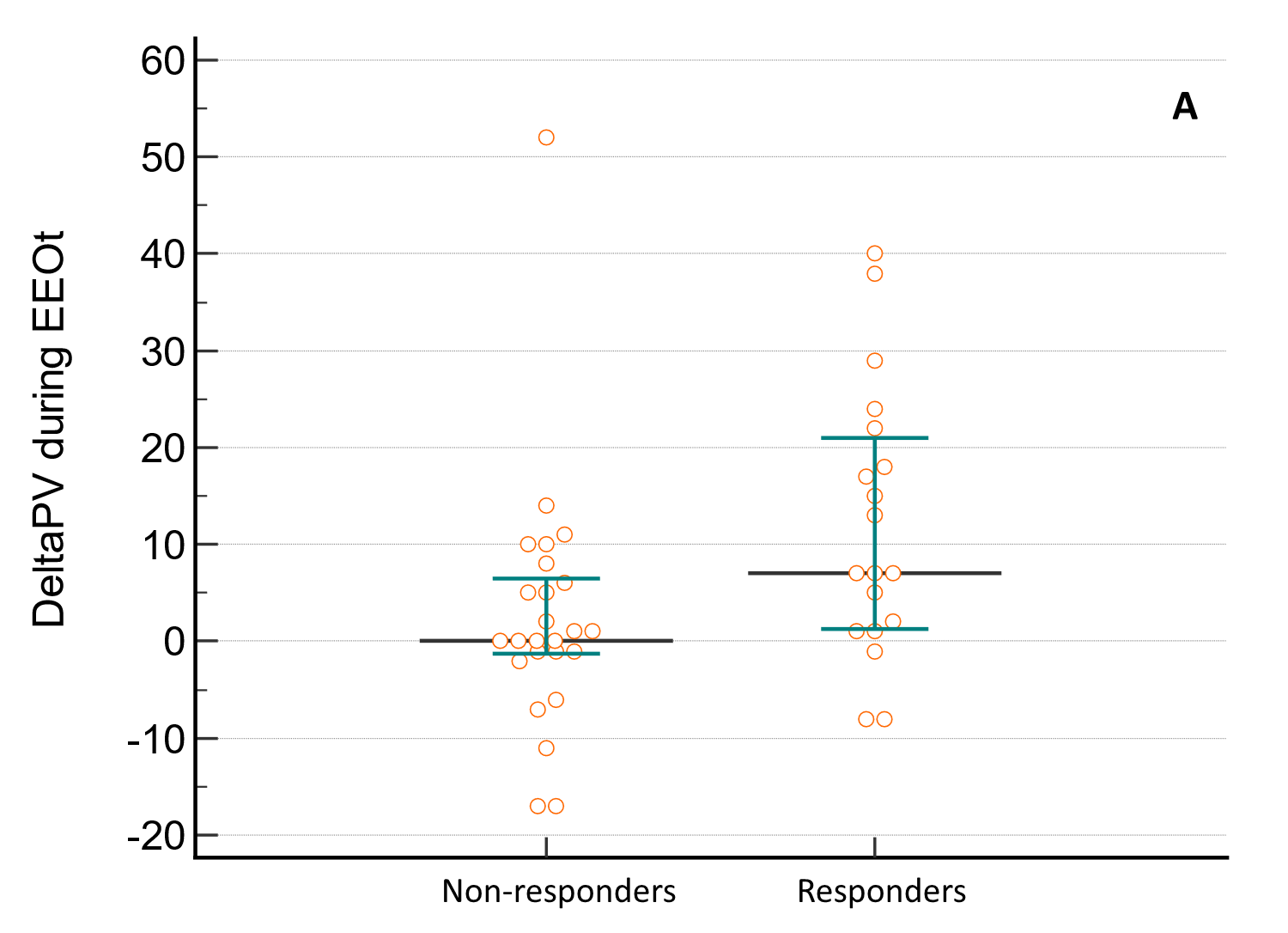


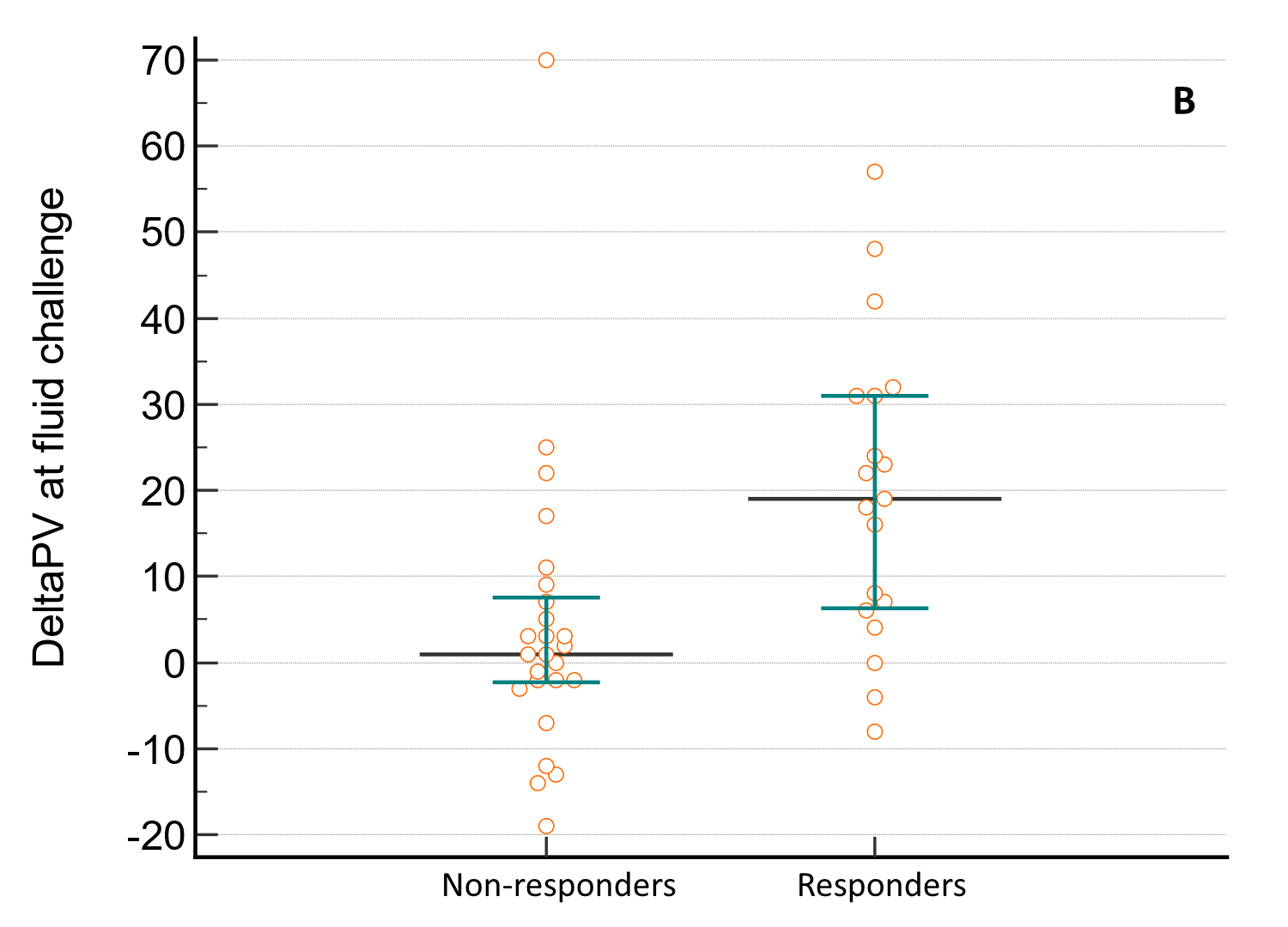


**ESM Figure E7**. Changes in CDPV during EEOt (**A**) and after fluid challenge (**B**) in non-responders and responders. Values are in cm/s. The black horizontal line is the median, while the upper and lower green lines represent the 25th and 75th percentile.
